# Application of Rank Annihilation Factor Analysis for Antibacterial Drugs Determination by Means of pH Gradual Change-UV Spectral Data

**DOI:** 10.3390/antibiotics9070383

**Published:** 2020-07-06

**Authors:** Mahnaz Esteki, Elham Dashtaki, Yvan Vander Heyden, Jesus Simal-Gandara

**Affiliations:** 1 Department of Chemistry, University of Zanjan, Zanjan 45195-313, Iran; elham.dashtaki@znu.ac.ir; 2 Department of Analytical Chemistry, Applied Chemometrics and Molecular Modelling, Center for Pharmaceutical Research (CePhaR), Vrije Universiteit Brussel (VUB), Laarbeeklaan 103, B-1090 Brussels, Belgium; yvanvdh@vub.ac.be; 3 Nutrition and Bromatology Group, Department of Analytical and Food Chemistry, Faculty of Food Science and Technology, University of Vigo—Ourense Campus, E-32004 Ourense, Spain

**Keywords:** bilinear matrix, pH-spectra, rank annihilation factor analysis, Sulfamethoxazole, Trimethoprim

## Abstract

The main objective of this study was to develop a simple and efficient spectrophotometric technique combined with chemometrics for the simultaneous determination of sulfamethoxazole (SMX) and trimethoprim (TMP) in drug formulations. Specifically, we sought: (i) to evaluate the potential use of rank annihilation factor analysis (RAFA) to pH gradual change spectrophotometric data in order to provide sufficient accuracy and model robustness; and (ii) to determine SMX and TMP concentration in drug formulations without tedious pre-treatments such as derivatization or extraction techniques which are time-consuming and require hazardous solvents. In the proposed method, the spectra of the sample solutions at different pH values were recorded and the pH-spectra bilinear data matrix was generated. On these data, RAFA was then applied to estimate the concentrations of SMX and TMP in synthetic and real samples. Applying RAFA showed that the two drugs could be determined simultaneously with concentration ratios of SMX to TMP varying from 1:30 to 30:1 in the mixed samples (concentration range is 1–30 µg mL^−1^ for both components). The limits of detection were 0.25 and 0.38 µg mL^−1^ for SMX and TMP, respectively. The proposed method was successfully applied to the simultaneous determination of SMX and TMP in some synthetic, pharmaceutical formulation and biological fluid samples. In addition, the means of the estimated RSD (%) were 1.71 and 2.18 for SMX and TMP, respectively, in synthetic mixtures. The accuracy of the proposed method was confirmed by spiked recovery test on biological samples with satisfactory results (90.50–109.80%).

## 1. Introduction

Sulfamethoxazole (SMX) and trimethoprim (TMP) are considered as effective antibiotics, which have been widely used for a long time. These two substances work by depriving bacteria of folate coenzymes [1]. The combination of sulfamethoxazole and trimethoprim, known as co-trimoxazole, is administered as various oral dosage forms, including tablets, syrups and suspensions, as well as human or veterinary intravenous infusions. This synergic combination produces a potent antibacterial agent, which is widely used for the treatment of a variety of ordinary bacterial infections, such as urinary tract infections [2], chronic bronchitis [3] and acute otitis media [4]. Therefore, due to the combined use of these two compounds in various drug formulations, the simultaneous analysis of SMX and TMP in drug and biological samples is of particular importance.

Several analytical methods, such as HPLC–UV [5,6], LC–MS [7] and capillary electrophoresis [8], have been developed to determine SMX and TMP in pharmaceutical formulations and biological fluids [9,10,11,12]. Most require relatively rigorous conditions; include serious sample-preparation steps which are expensive, time consuming and need hazardous solvents; and costly in terms of required instrumentation and well-trained technicians. Moreover, ordinary analytical laboratories are not capable of performing these methods. Therefore, one can consider the development of accurate and reliable methods, focusing on an easy simultaneous determination of SMX and TMP, using simple and low cost instrumentation, as an appropriate alternative purpose [13,14]. 

UV–Vis absorbance spectrophotometry is considered a convenient and accessible technique for quantitative analysis, which can be applied as a rapid, sensitive and cost efficient tool. Furthermore the main advantages of this technique are being simple, precise and accurate [15]. Nonetheless, the low selectivity and specificity are counted as the UV–Vis spectrophotometry weaknesses, which are due to the spectral overlap of various components. This makes the use of chemometric techniques on the resulting data of interest [16].

The univariate calibration is then definitely inefficient due to the absence of analyte-selective information, which is caused by the presence of interferents. Consequently, multivariate calibration techniques are frequently employed to the quantification of multi-component mixtures using spectrophotometric methods [17,18]. Multivariate calibration modeling provides the opportunity to extract selective signals from the none-selective measured data through mathematical approaches. Due to the fact that the signal profile of the interferences is not absolutely identical to the signal of the analyte, interferents can be mathematically eliminated [19]. 

Chemometric techniques, such as multicomponent derivative spectroscopic analysis [20], ratio spectra derivative spectrometry [21] and bivariate calibration spectrophotometry [22], have also been employed to determine SMX and TMP simultaneously. These methods are mainly first-order calibration techniques that suffer from unknown interferences, denoting that, in the case of the presence an unknown interference inside the sample matrix, analysis of the real samples would not be accurate. Accordingly, nowadays, special attention has been paid to high-order data, to overcome this drawback. These data, which have been generated in different analytical chemistry fields, are provided by a wide range of up to date instruments, mainly applicable to multicomponent analysis in complex matrices. Specifically, second-order (two-way) data, in which a single sample generates a data matrix, are increasingly gaining attention [23,24]. Particularly, the uniqueness of the decomposition of a three-way data array attribute to second-order data makes it very interesting to be applied. This enables us to extract the concentrations and spectral profiles of individual components without interference from uncalibrated absorbing components. In other words, second-order calibration is capable of offsetting the potential interferences that are not represented in the calibration set. Accordingly, it is possible to determine accurately the concentrations of active components present in a sample. This property, known as the “second-order advantage”, can be considered as an attractive alternative with significant potential for the multi-component analysis, which avoids the requirement of interference removal from complex mixtures [25]. The three-way data can be produced by using hyphenated methods, although they can also be obtained through a single instrument, such as excitation–emission fluorescence and absorbance–time measurements [23]. In the latter case, the third dimension of the data may be reaction time or pH gradient. The absorbance changes due to variations in the proton-transfer species composition caused by pH alteration give rise to an absorbance spectra–pH data matrix. A three-way array can be produced by measuring these data matrices for a set of samples [26].

Rank annihilation factor analysis (RAFA) is a chemometric technique for multicomponent calibration based on rank analysis of two-way array data that can be used for the quantitative analysis of a sample with unknown background. Reducing the rank of the unknown sample by subtracting the contribution of the analyte present in a standard matrix is the basis of the method. The proposed method yields a new data matrix with a rank decreased by one unit through subtracting the signal from the analyte of interest from the sample data, because of the fact that for ordinary bilinear rank-one data, such as spectrophotometric or fluorescence data, the rank of the analyte of interest is one [27]. RAFA has been applied as a second-order calibration method for qualitative and quantitative analysis in different fields [28,29,30,31]. 

The main objective of this study was to develop a simple and efficient spectrophotometric technique combined with RAFA for the simultaneous determination of SMX and TMP in drug formulations. pH gradual change spectrophotometric data were used to achieve the second-order advantage data, making it possible to determine SMX and TMP concentration in drug formulations without tedious pre-treatments such as derivatization or extraction techniques which are time-consuming and require hazardous solvents. The potential of applying RAFA to pH gradual change spectrophotometric data was evaluated to provide sufficient accuracy and model robustness. 

To the best of our knowledge, there is no report in the literature on the determination of SMX and TMP based on the absorbance differences of the analytes at different pH values. In this work, we propose a simple, inexpensive, sensitive and selective procedure for direct spectrophotometric determination of SMX and TMP in pharmaceutical and biological samples by implementing RAFA to pH-gradual-change UV-spectral data. The proposed method can predict the concentration of SMX and TMP (regarding their linear dynamic range, with concentration ratios of SMX to TMP varying from 1:30 to 30:1 in the mixed samples) in different samples with satisfactory results.

## 2. Materials and Methods

### 2.1. Instruments

Spectrophotometric measurements were made with a SHIMADZU dual beam UV–Vis spectrophotometer; model UV-160 PC (Kyoto, Japan). The absorbance spectra of the reference and the test solutions were recorded in 1.0-cm quartz cells over the range 200–350 nm at room temperature. A Metrohm 780 digital pH meter (Herisau, Switzerland), Soronex Digitec DT 102H ultrasonicator (Bandelin, Germany) and a universal centrifuge, model premium 20,000 R (Iran), were used in the experiments. All chromatographic measurements were made with a Waters 1500 Series instrument (Milford, MA, U.S.A.) equipped with dual absorbance detector (Waters Model 2487). The separation was carried out on a reversed phase C18 HPLC column (5 µm, 4.6 mm × 250 mm; ODS, Merck, Darmstadt, Germany). The details of the HPLC experiments for determination of SMX and TMP have been explained elsewhere [32]. Experimental data were processed with programs written in MATLAB (version 6.5, Mathworks, Natick, MA, USA).

### 2.2. Chemicals and Reagents

SMX and TMP were provided from Sobhan Darou Company (purity > 98%) (Rasht, Iran). All other chemicals and solvents used in the experiments were of analytical grade. All solutions were prepared with deionized water (>18 MΩ) which was made in house using TKAsmart2pure Water Purification Systems (Niederelbert, Germany). Pharmaceutical samples analyzed in this work were obtained from local drugstores. The quantities of two drugs in these pharmaceutical products, which were mentioned by producer companies, were considered as approximate quantities.

Human serum and plasma samples were prepared from Vali-asr hospital, Zanjan, Iran. All donated blood and plasma samples have the informed consent of patients for research uses and were coded to avoid binding to a specific patient in accordance with standard Vali-asr hospital procedures, which complies with the requirements of protection of data according to the Directive of Iran National Committee for Ethics in Biomedical Research (https://ethics.research.ac.ir/upload/5qeg3fqbtgvwx38z.docx).

### 2.3. Standard Solutions

Primary stock solutions of SMX and TMP (all at 1000.0 µg mL^−1^) were separately prepared by dissolving 10.0 mg of each standard powder in the least amount of acetonitrile and completed to volume with deionized water. Suitable amounts of primary stock solutions of SMX and TMP were transferred to 25.0 mL volumetric flasks followed by the addition of 5.0 mL of the Britton–Robinson buffer solution (0.1 mol/L) and diluted with water to prepare standard working solutions (1, 5, 10, 15, 20, 25 and 30 µg mL^−1^ for both SMX and TMP). All solutions were stored at 4 °C and equilibrated to room temperature before use. The working standard solutions for spike recovery were also prepared at required concentrations (10, 20 and 30 µg mL^−1^ for SMX and 10, 20 and 25 µg mL^−1^ for TMP) from the stock standard solutions.

### 2.4. Treatment of Real Samples

Drug samples of co-trimoxazole, i.e. adult co-trimoxazole tablet, pediatric co-trimoxazole tablet, co-trimoxazole oral suspension and co-trimoxazole intravenous infusion, containing different amounts of SMX and TMP, were used as real samples of pharmaceutical formulations. For tablet samples, 10 tablets were weighed and powdered, and then a proper quantity of powder was dissolved in acetonitrile and diluted with water in a 250 mL volumetric flask. For intravenous infusion samples, 10 ampoules were combined in addition to dissolving and diluting with water in a 250 mL volumetric flask. Further diluted solutions were also made by serial dilution with water. 

Human serum and plasma samples were carefully collected and kept in frozen conditions until analysis. Then, 1.0 mL of the sample was pipetted into a centrifugation tube that contains known concentrations of SMX (10, 20 and 30 µg mL^−1^) and TMP (10, 20 and 25 µg mL^−1^). Then, 10.0 mL of methanol was mixed properly with the solution for protein precipitation. The solution was subjected to a centrifugal force of 4000 rpm for 10 min, in order to separate the precipitated proteins. The process continued by passing the clear supernatant layer through a 0.45 µm Millipore filter. The percolated solution was collected and transferred into a volumetric flask of 25.0 mL followed by adding 10 mL of Britton–Robinson buffer solution (with considered pH) and completed to volume with deionized water and then the resulting solution was introduced to the spectrophotometer cell.

### 2.5. Spectrophotometric Analysis of Sulfamethoxazole and Trimethoprim in Laboratory-Prepared Mixtures 

The procedure of preparing solutions containing various proportions of analytes (Table 1 and Table 2) started with precisely pipetting certain amounts from their standard working solutions into a sequence of volumetric flasks of 25.0 mL, proceeded by addition of 10.0 mL of the Britton–Robinson buffer solution (with considered pH), and bringing to volume with water. The final concentration ranges were 1.0–30.0 µg mL^−1^ for both SMX and TMP. The absorbance spectra, from 200 to 350 nm, of these laboratory-prepared mixtures were recorded. Concentrations of the analytes in the prepared samples were estimated from the corresponding RAFA models using standard solutions of 5.0 µg mL^−1^ SMX and 5.0 µg mL^−1^ TMP.

### 2.6. Theory of Rank Annihilation Factor Analysis

Rank annihilation factor analysis (RAFA) [33] is a technique invented to assay a known analyte which is accompanied by unknown interferents in a sample. The basis of RAFA is that eliminating the exact amount of the analyte signal from a mixture data matrix results a matrix rank reduction. The numerical rank of a matrix specifies the number of linearly independent column vectors or rows of the matrix. Considering the presence of noise, the number of rows or columns (the smaller number) in the data provides the mathematical rank of an absorbance matrix. Accordingly, the chemical rank as the number of relevant chemical factors that may be extracted from the data matrix is more interesting in analytical chemistry. The chemical rank can be estimated by a variety of existing methods giving an approximation for this number [34]. 

Supposing that **R** indicates the residual matrix, **S** the single component standard matrix and **M**, the mixture matrix, the following equation can be written:

(1) R=M−k.S

The main issue regarding this equation is to find the proper value for parameter k so that the rank of **R** is one less than of **M**, by which the contribution of the single component should be removed from matrix **M**. Hence, the concentration of the component in a mixture can be estimated using the following equation:

(2) $$C_{x}=C_{S}\times K$$

where C_s_ and C_x_ are the component concentrations in standard and mixture solutions, respectively. To select the parameter k properly, principal component analysis (PCA) was applied iteratively to the residual matrix (**R**) resulting in different values of k on each run, for which the RSD (Relative Standard Deviation) was assessed afterward. The RSD is an assessment of principal component model fit deficiency for a dataset. As far as the residual matrix RSD value reaches its minimum, decomposition of matrix **R** (Equation (3)) is required to achieve the optimal solution. The RSD is calculated as stated below [35]:

(3) $$RSD\left(n\right)={\left(\overset{c}{\underset{i=n+1}{\sum }}\frac{g_{i}}{n\left(c-1\right)}\right)}^{1/2}$$

In this equation, *g_i_* represents the eigenvalue, *n* is the number of principal components and *c* is the rank of the data matrix. 

### 2.7. Chemometrics Models

A two-factor, five-level full factorial design was built using each pair of components with 5 varying concentrations coded from −2 to +2 to be sure about the robustness of the model. Applying this approach, we should consider five concentration levels for each compound, resulting in 25 mixtures in the following concentration ranges: 1.0–30.0 µg mL^−1^ for SMX and 1.0–30.0 µg mL^−1^ for TMP. The concentrations of all considered levels for each compound are based on the design requirements. All mixtures of this design were used as a validation set to test the predictive capability of the developed multivariate RAFA model that was built by using standard solutions of 5.0 µg mL^−1^ SMX and TMP. All spectra of mixtures were mean-centered, in preparation for modeling. RAFA was carried out using a laboratory-created program, routinely implemented in MATLAB 6.5.

## 3. Results and Discussion

The main goal of this study was to establish a low-cost, simple, sensitive and accurate analytical method for the simultaneous determination of SMX and TMP in their mixtures, drug formulations and biological fluids, as well as to construct a model with satisfactory accuracy for effective analytical practice. 

### 3.1. Linear Calibration Models for A Single Component 

Calibration curves were plotted to find the linear dynamic range of each component. The absorbance spectra were recorded in the range 200–350 nm relative to a solvent blank. Plotting the absorbance at its λ_max_ (SMX, 254.5 nm; TMP, 282 nm) vs. sample concentration gives the linear range for the considered component. Table 1 illustrates the calibration models and the respective figures of merit. Linear dynamic ranges (LDRs) were 1.0–30.0 µg mL^−1^ for both compounds and the coefficients of determination (*R*^2^) were 0.994 and 0.996 for SMX and TMP, respectively. Detection limits were achieved equal to 0.25 and 0.38 µg mL^−1^ for SMX and TMP, which are proper values for analysis of drugs. In this research, performing a five-level full factorial design (comprising 25 solutions) made it possible to select a set of mixtures covering the entire experimental domain. The contribution of components was assured to be additive and in accordance to the linear range of the spectrophotometer. Table 2 shows the actual and predicted concentrations of SMX and TMP in synthetic mixtures. As can be seen, the accuracy of the results is satisfactory in all cases, when the concentration ratio of SMX and TMP vary from 1:30 to 30:1. The RSD values are all <4.0%, which shows the reproducibility of the method.

**Figure 1:**
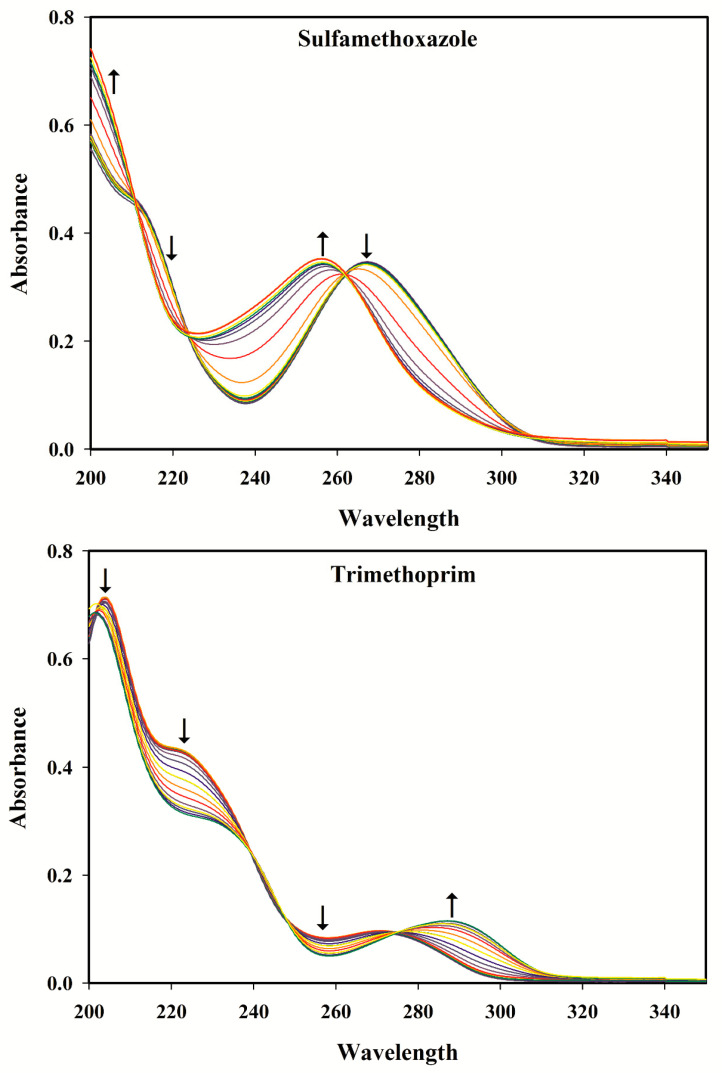
UV absorbance spectra of sulfamethoxazole (5.0 µg mL^−1^) and trimethoprim (5.0 µg mL^−1^) at different pHs (pH from 2 to 12 at intervals of 0.5).

**Figure 2:**
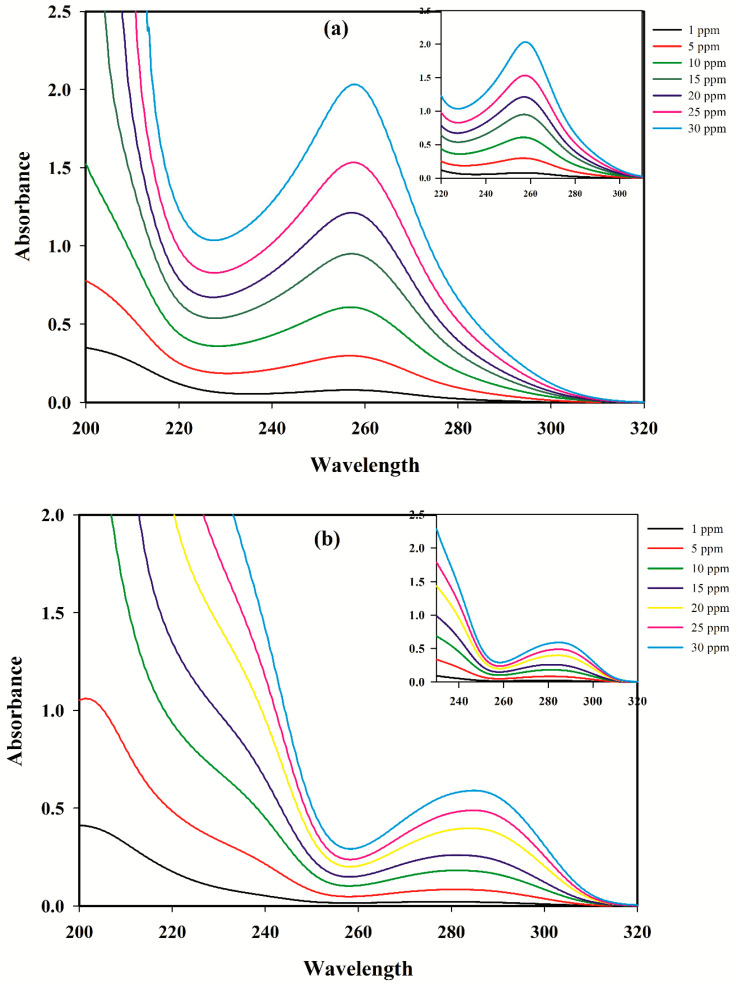
UV absorbance spectra of (**a**) sulfamethoxazole (pH = 5.4) at different concentrations (1–30 µg mL^−1^); and (**b**) trimethoprim (pH = 7.0) at different concentrations (1–30 µg mL^−1^).

The absorbance spectra of the two analytes recorded in the range of 200–350 nm are shown in Figure 1 and Figure 2. A strong spectral overlap is observed, which complicates the individual determination of the compounds from the spectrum of a mixture.

### 3.2. Selection of the pH Range

Figure 1 shows the spectra of SMX and TMP in different pH buffer solutions (pH 2–12). By increasing pH from 2 to 12 at intervals of 0.5, a hypsochromic shift takes place for SMX, whereas a bathochromic one can be observed for TMP. These results are in agreement with a previous report by Zhou & Moore [36]. Sulfamethoxazole is an acidic compound and its spectrum undergoes a hypsochromic shift with increasing pH, which is related to the loss of a proton from the -SO_2_-NH- group. Trimethoprim is a basic compound and a proton is associated with the NH_2_ substituents in acidic solution, but the bathochromic shift occurs as the pH is increased [36]. Additionally, there is no significant variation between the spectra recorded at pH 8 to 12 for SMX. For TMP, however, no considerable change was observed in the pH range 9 to 12. Therefore, it seems that there is no considerable information in pH region 9–12, thus the range 2–9 (including 2.0, 2.5, 3.0, 3.5, 4.0, 4.5, 5.0, 5.5, 6.0, 6.5, 7.0, 7.5, 8.0, 8.5 and 9.0) was selected for the determination of SMX and TMP. 

### 3.3. pH-Spectral Absorbance Data: Bilinearity, Trilinearity and Rank Deficiency

To perform multi-component calibration, RAFA utilizes two data matrices simultaneously; the first matrix is composed of the unknowns and the second of calibration samples. The rank annihilation technique would entail linear and additive measured signals; the constructed data in this way are called bilinear. Rank annihilation requires the signal for the intended analyte to be identical for all samples, as well as to be independent of the remaining substituents signal [37]. The data collected from a given concentration of SMX (and/or TMP) are composed of absorbance spectra at consecutive pH values, with the absorbance values recorded at n wavelength points (200–350 nm at intervals of 0.5 nm, 301 points) in each spectrum. Then, it is possible to arrange the spectra of the solution at a variety of pH values to form a data matrix **D**, made up of n columns (301 wavelength points) and m rows (15 pH points), representing the number of wavelengths and pH values, respectively. The following equation describes the application of Lambert–Beer law to obtain the matrix **D**:

(4) $$D={\mathrm{CS}}^{T}$$

where the dimensions of these matrices are **D** (m × n), **C** (m × r) and **S** (n × r), and the superscript T is an indication of a transposed matrix. Matrix **S** comprises particular spectra of the different chemical forms of SMX (or TMP), while matrix **C** integrates the concentrations of these forms at different pH values. Consequently, matrix **D**, with n rows and p columns, is a bilinear pH–spectra matrix. 

As discussed above, various types of second-order data constructed by different methods, can be calibrated through second-order calibration techniques to quantify target analyte(s) accurately, even if there are unmodeled or unexpected interferents in the mixture [26]. To obtain a robust calibration model and take advantage of the second order, a true trilinear dataset should be utilized as starting point [26,38]. If the pure analyte response shows the same form in both standard and unknown mixtures, then the joint variation in the standard and unknown mixtures can be modeled using trilinear models [39].

Regarding the chemical aspects, the concentration–absorbance–pH data that are produced for acid/base species necessarily should be trilinear. It should be mentioned that second-order calibration models would be considerably limited, if applied to the data deviating slightly from trilinearity [40]. In most cases, the primary reason for such deviations are changes in the peak shapes [40]. Figure 2 shows the spectral profile of SMX and TMP in their linear concentration range from 1 to 30 µg mL^−1^. Considering the shape of the spectra of the two analytes in different concentrations (from 1 to 30 µg mL^−1^), it seems that altering the concentrations of SMX and TMP results in small changes below 220 nm and above 320 nm. Therefore, to meet the trilinear conditions, only the spectral interval between 220 and 320 nm was selected for further chemometric analysis.

Capitalizing on the analyte’s acidic/basic characteristics, we can choose the pH as parameter to modulate the second data dimension. In these situations, the generated data are linearly dependent on the pH profiles and the concentrations of proton-transfer species are correlated [41]. When more than one analyte is present, the aforementioned dependency gives rise to rank-deficiency, which means that the comprehensive rank of the measured data and the sum of the ranks of the individual species contribution do not yield the same value [41]. This problem is observed under conditions that the number of independent reactions is smaller than the number of response-active species, i.e. when the number of independent components is lower than the number of real chemical components present in the system. The rank deficiency is definitely one of the significant sources of deviation from trilinearity. However, rank deficiency can be eliminated deploying a matrix augmentation method, which is the simultaneous analysis of the corresponding matrix along with one additional full rank standard matrix. Some authors [42] have discussed the primary concept of the augmentation effect on solving the rank deficiency. Based on this explanation and regarding the abovementioned conditions in which the problem has to be solved, rank deficiency removal was performed using column-wise augmentation together with a standard data matrix. Because of this method implementation, an improved resolution of the system would be achieved.

### 3.4. Rank Analysis

**Figure 3:**
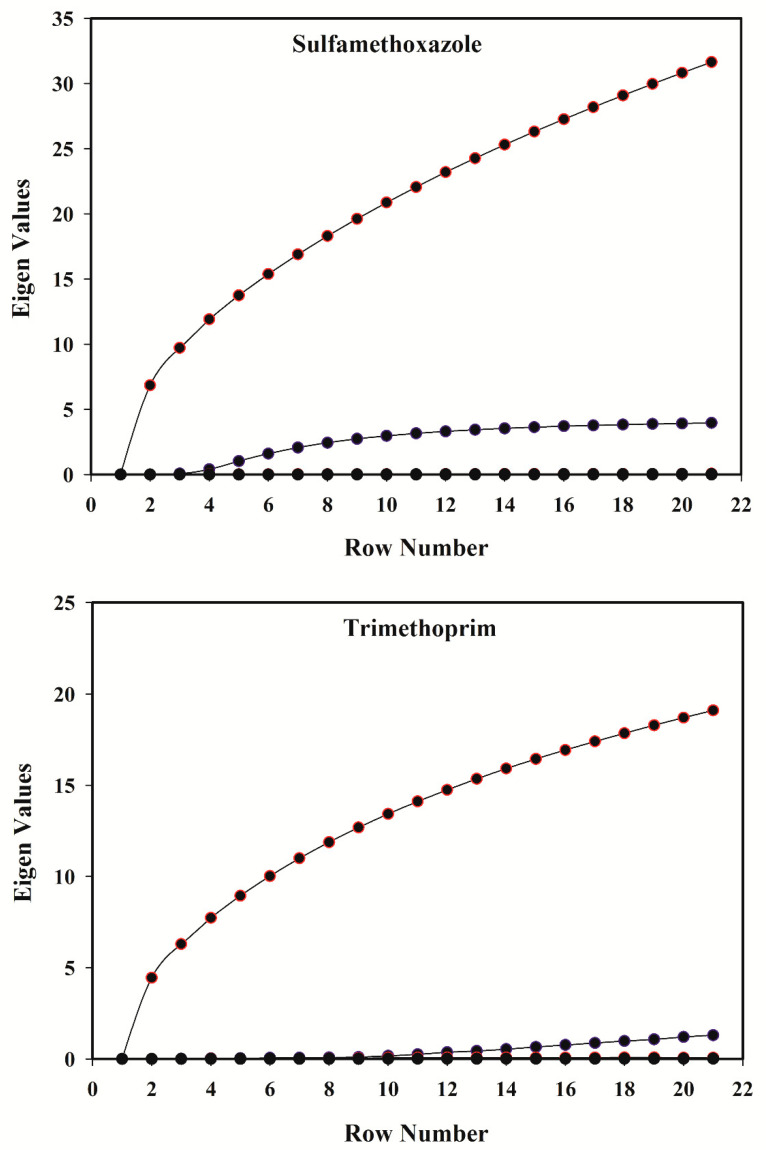
Eigenvalue vs. row number in Evolving Factor Analysis to determine the number of species contributing to the spectral signal of sulfamethoxazole and trimethoprim standard sample solutions.

**Figure 4:**
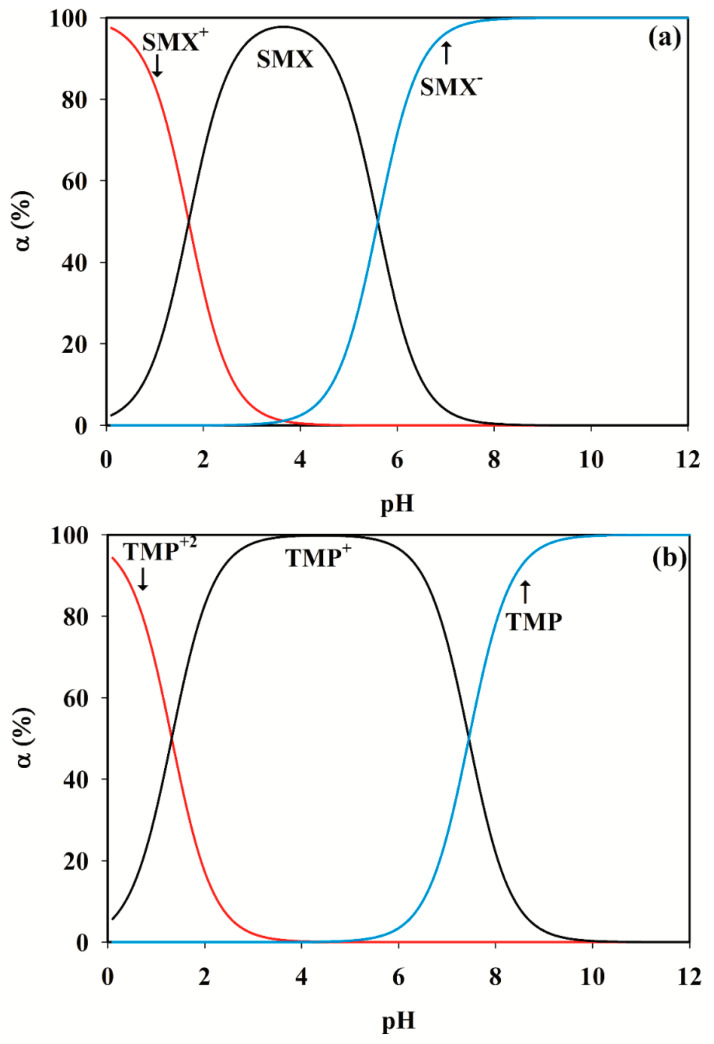
Distribution of species at different pHs for: (**a**) sulfamethoxazole; and (**b**) trimethoprim.

**Figure 5:**
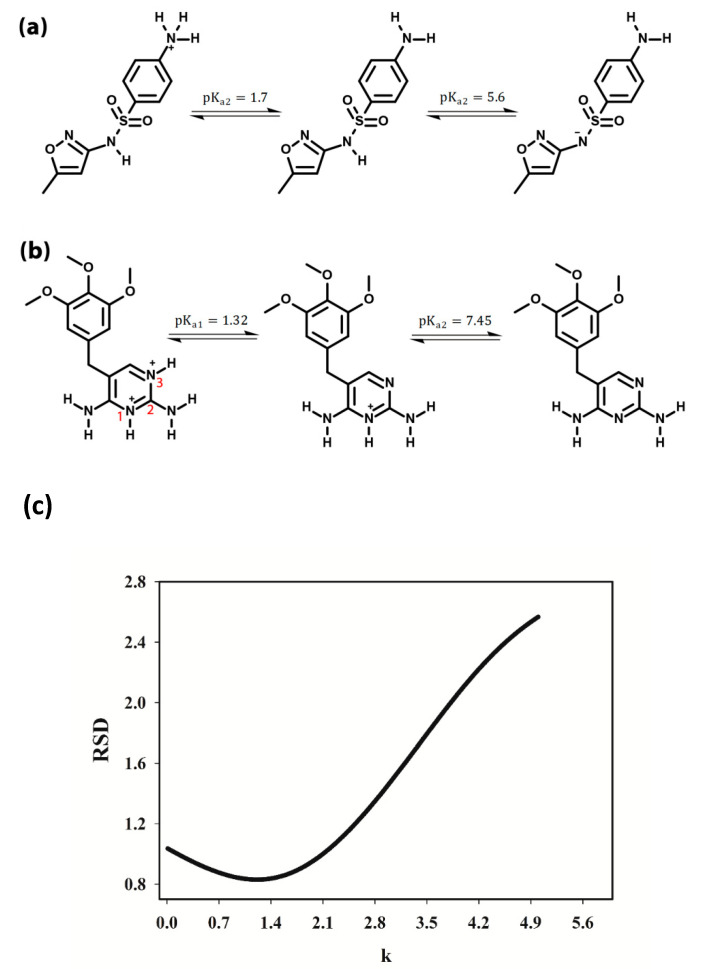
The structures of sulfamethoxazole (**a**) and trimethoprim (**b**) at different pH values; and (**c**) RSD values obtained for parameter k values in RAFA analysis.

In this study, Evolving Factor Analysis was used to determine the number of species contributing to the spectral signal [43]. This method offers the opportunity to determine the number of chemical components, through investigating the eigenvalues of the submatrices, resulting from spectroscopic data that are arranged in ascending order of the evolutionary variable (pH). Considering only the initiating spectrum, adding successive spectra to the previous sub-matrix gives rise to a succession of sub-matrices, of which the eigenvalues are extracted, normalized to unit sum, and finally their logarithms are plotted as a function of the sequence number. Visual inspection of the plot gives an indication of real factor appearance above the noise level. Based on this method, by employing the singular value decomposition (SVD) algorithm, it is possible to achieve the matrix rank by calculating the eigenvalues of the first row, then the first and the second row, and so on until all rows. Finally, the eigenvalues that are obtained in each step are plotted. The number of results higher than the noise level implies the rank of the matrix. Figure 3 illustrates the eigenvalues regarding each row of the given data matrices and shows the rank of the single-solute solutions of both SMX and TMP equals 2. This can be justified regarding existing species in the standard solution of SMX and TMP, at different pH values. The pK_a_ values of these two compounds, besides the different forms of the species in acidic and basic solutions, are shown in Figure 4 and Figure 5. 

As mentioned by Babić and Chen [44,45], observing previously performed studies, an agreement about the SMX molecular forms at different pH values exists. As depicted in Figure 5, SMX has a sulfonamide group with pKa value of 5.6 and an amine attached to the aromatic ring, with a pKa of 1.7 (referred to as N1 and N4, respectively). Between 1.7 and 5.6, the molecules are not neutral. One could state that, to have only neutral SMX for the amine group, pH should be above 4.7 (pKa + 3). However, at this pH, about 10% of the sulfonamide group is already negatively charged. The same reasoning is valid for the sulfonamide group (uncharged below 2.6 (pKa − 3), but then 10% of amine groups is already charged. The equilibrium concentrations of different forms of SMX at various pH values can be achieved using the following equilibrium relations, considering that

[SMX]_tot_ = [SMX^+^] + [SMX] + [SMX^−^]. 
SMX + H_2_O ⇆ SMX^−^ + H_3_O^+^
SMX^+^ + H_2_O ⇆ SMX + H_3_O^+^

where SMX^−^ is an SMX molecule which has lost its proton, SMX^+^ is an SMX molecule which has gain a proton and SMX represents a neutral SMX molecule.

Figure 4 depicts the presence of the three absorbing compounds in the pH range of 2–12 that we considered in this work. 

The discussion about TMP, however, is somehow controversial. Firstly, in many references, only a single *pK_a_* value of about 7 has been taken into account [46,47], while TMP actually has two *pK_a_* values, *pK*_*a*1_ and *pK*_*a*2_, which are about 1.32 and 7.45, respectively. Secondly, different descriptions of the TMP protonation process in acidic pH ranges makes this compound more challenging to observe. The protonation of TMP is demonstrated as proton absorption by an amino group in some references (e.g., [48]); nonetheless, the study of the results obtained by NMR spectroscopy [49] and capillary zone electrophoresis [50] define it as a two-step process involving two heterocyclic nitrogen atoms (N1 and N3) (see Figure 5). 

The hydrogen bonding analysis and molecular packing of TMP and other comparable compounds, using computational methods and crystal structure determinations, were carried out [51]. Others have claimed that the favored site for protonation is on the N1 (ring nitrogen) [50], leading to *pK*_*a*2_ values between 6 and 7 and full protonation at pH 2.1. As for most nitrogen heterocycles, *pK*_*a*1_ values are likely to be near 1 or 2 [52], and protonation of TMP is supposed to occur at the N3 position [51].

Accordingly, at different pH values, the following equilibriums are established:

TMP^2+^ + H_2_O ⇆ TMP^+^ + H_3_O^+^*pK*_*a*1_ = 1.35
TMP^+^ + H_2_O ⇆ TMP + H_3_O^+^*pK*_*a*2_ = 7.45

where TMP^+^ is a TMP molecule which has gain a proton, TMP^2+^ is a TMP molecule which has gain two protons and TMP represents a TMP neutral molecule)

Based on these equilibrium equations and considering that [TMP]_tot_ = [TMP^2+^] + [TMP^+^] + [TMP], the concentration of different TMP forms at various pH values can be obtained. The results of these computations are shown in Figure 4. Thus, at pH values lower than *pK*_*a*1_, both nitrogen rings would be protonated (TMP^2+^), while, at the pH range between *pK*_*a*1_ to *pK*_*a*2_, a proton is being released to form the TMP^+^. Finally, at pH values above *pK*_*a*2_, TMP loses the second proton and the compound becomes neutral (TMP).

Spectrophotometric analysis based on pH modulation provides rank-deficient data matrices for mixture of components with acid–base behavior [53]. If the number of significant factors contributing to the data variances, which is determined through singular value decomposition or other factor analysis approaches, is lower than the real number of existing chemical components in the signal contribution, the data matrix is supposed to be rank deficient. For closed-reaction systems, such as those studied here, the total concentration of the mixture is constant during pH alteration for each of the acid–base pair components, which implies mixtures with a lower number of independent reactions than response-active absorbing species.

Therefore, it is clear why the graphs in Figure 3 determine rank two for a standard solution including SMX (or TMP), under conditions in which there are three absorbing species in the current system. When changing the pH is used for modulating the second data dimension, concentrations exhibit linear dependencies on the pH profiles, which is the consistency of the aggregate individual proton-transferring species. For a system containing more than one analyte, this dependency would lead to rank-deficiency, which means that the overall rank of the measured data is not equal to the sum of the individual species contribution ranks [42]. Therefore, it is obvious that the rank of a mixture including SMX and TMP is determined as 3, whereas the individual summation of rank contribution of SMX and TMP equals 4. 

### 3.5. Determination of SMX and TMP in Validation Samples

Given a spectrum, which is the result of different components absorbance, rank annihilation factor analysis (RAFA) gives us the opportunity to quantify a specific component contributing to the spectrum, while the quantification and identification of the other components is not necessary. The performance of RAFA in SMX and TMP determination, in both validation samples and pharmaceuticals, was carried out. The possibility of uncalibrated and unexpected interferents existence in the samples is also noteworthy. In addition to the preparation of a 5 µg mL^−1^ standard single-solute solution of SMX (or TMP) for building the model, multicomponent solutions with a wide range of SMX and TMP concentrations were also made to evaluate the performance of the model. As mentioned in Section 3.2, calibration was carried out deploying three PCs to build the model for both SMX and TMP determination. 

A two-way data matrix was constructed by measuring the absorbance under different conditions in terms of wavelength and pH values, while the concentration of the analyte was kept constant. 

As mentioned above, the RAFA methodology entails two bilinear datasets, **S** and **M**, which represent the calibration standard set and the sample set, respectively. As discussed in Section 3, the first step is to estimate the rank of **R**, which equals **M**-k.**S**, using SVD. Then, an iterative procedure, plotting the eigenvalues (or singular values) of least significant PCs of **R**, is applied as a function of k to find the minimum value of k. Figure 5c gives an example of finding minimum k-value for determining the concentration of the analyte in the mixture, here with the k-value 1.21. The concentration of the intended analyte in the calibration standard was 5.0 µg mL^−1^; therefore, the anticipated concentration of this analyte in the unknown sample was determined as 6.05 µg mL^−1^. 

The analysis of the synthetic mixtures was carried out for the measurement of the prediction error, which brought about the results illustrated in Table 1 and Table 2, representing individual errors less than 5.6% and 3.0% for the calculated overall prediction error. 

### 3.6. Determination of SMX and TMP in Real Samples

To evaluate method performance in the simultaneous determination of SMX and TMP, a proficiency test was carried out by analyzing some pharmaceutical formulations (Section 2.3) and biological fluids. The results of this test are shown in Table 3 and Table 4, demonstrating a good agreement between the results achieved from the analysis of pharmaceutical formulations and those obtained through an HPLC method. 

Furthermore, the method performance was tested on some biological fluids (human serum and plasma), and the spiked standard solutions verified the obtained results with high consistency (Table 3 and Table 4). Comparison of the results (percent recovery) of the proposed method with the HPLC demonstrated the robustness of the proposed method. 

## 4. Conclusions

In this study, a straightforward and inexpensive approach was developed for the simultaneous detection of SMX and TMP in the pH-spectral matrices, employing RAFA method. The spectra of the sample solutions at different pH values were recorded and the pH–spectra data matrix was created. RAFA was then applied on these data to determine the concentrations of SMX and TMP in synthetic and real samples. The obtained results show that the two compounds could be determined simultaneously with concentration ratios of SMX to TMP varying from 1:30 to 30:1 in the mixed samples (concentration range is 1–30 µg mL^−1^ for both components). The presented method was also capable of a precise determination of SMX and TMP in pharmaceutical formulations and biological samples with high recoveries. The principal advantages of this approach over other techniques are: (1) the complicated operation of pre-separation can be omitted; (2) it is easy to produce second-order data using this approach; and (3) the required equipment in this technique is widely available. We may also take advantage of this method for SMX and TMP determination in other types of samples, such as urine and wastewater. However, the main discussion of this paper only concerns the method application for determination of SMX and TMP in some biological and pharmaceutical samples.

## Figures and Tables

**Table 1:** Calibrations and figures of merit for sulfamethoxazole (SMX) and trimethoprim (TMP).

Parameters	Sulfamethoxazole	Trimethoprim
Linear range (µg mL^−1^)	1.0–30.0	1.0–30.0
Correlation coefficient	0.994	0.996
Intercept	−0.0196 ± 0.0033 (*n* = 3)	−0.0121 ± 0.0025 (*n* = 3)
Slope (mL^−1^ µg)	0.0632 ± 0.0028 (*n* = 3 )	0.0197 ± 0.0020 (*n* = 3)
Detection limit (µg mL^−1^)	0.25	0.38

**Table 2:** The concentrations of SMX and TMP and the results of the replicate measurements for determination of SMX and TMP in synthetic mixtures.

Sample No.	SMX				TMP			
	Actual (µg mL^−1^)	Predicted (µg mL^−1^)	Error (µg mL^−1^)	RSD (%, *n* = 3)	Actual (µg mL^−1^)	Predicted (µg mL^−1^)	Error (µg mL^−1^)	RSD (%, *n* = 3)
1	1	0.91	−0.09	3.1	1	1.03	0.03	2.78
2	6	5.65	−0.35	2.32	1	0.98	−0.02	2.12
3	13	14.59	1.59	1.51	1	0.95	−0.05	2.98
4	20	20.18	0.18	3.20	1	1.08	0.08	3.15
5	30	30.55	0.55	0.55	1	0.96	−0.04	3.19
6	1	0.89	−0.11	1.68	6	5.66	−0.34	1.59
7	6	6.58	0.58	2.32	6	6.12	0.12	2.31
8	13	12.77	−0.23	1.98	6	6.23	0.23	1.66
9	20	19.32	−0.68	1.33	6	5.89	−0.11	1.45
10	30	28.51	−1.49	2.52	6	6.08	0.08	1.98
11	1	1.05	0.05	2.31	13	13.78	0.78	1.32
12	6	6.87	0.87	1.56	13	12.32	−0.68	1.75
13	13	13.88	0.88	1.23	13	12.42	−0.58	2.13
14	20	21.89	1.89	1.32	13	13.88	0.88	1.55
15	30	29.12	−0.88	1.45	13	13.59	0.59	1.32
16	1	1.10	0.1	1.05	20	20.13	0.13	2.62
17	6	5.64	−0.36	1.23	20	21.16	1.16	1.78
18	13	14.02	1.02	2.12	20	19.55	−0.45	3.21
19	20	22.21	2.21	1.21	20	19.17	−0.83	2.17
20	30	28.65	−1.35	1.59	20	21.21	1.21	2.79
21	1	0.99	−0.01	2.32	30	32.42	2.42	1.98
22	6	5.22	−0.78	1.48	30	30.67	0.67	3.31
23	13	14.65	1.65	1.75	30	29.10	−0.9	2.78
24	20	20.03	0.03	0.75	30	31.78	1.78	1.44
25	30	31.22	1.22	0.89	30	32.52	2.52	1.32

**Table 3:** Results of the replicate measurements (*n* = 3) for the determination of SMX and TMP in some pharmaceutical formulations.

Drug	SMX			TMP		
	Approximate Doses (mg)	Proposed Method (mg)	HPLC (mg)	Approximate Doses (mg)	Proposed Method (mg)	HPLC (mg)
Co-trimoxazole adult tablet	400	403 ± 4.3	402 ± 5.2	80	78.3 ± 2.6	79.6 ± 3.1
Co-trimoxazole pediatric tablet	100	98.23 ± 3.7	97.3 ± 4.1	20	22.3 ± 3.1	24.8 ± 4.1
Co-trimoxazole oral suspension	200	197.56 ± 4.9	198.4 ± 4.7	40	38.5 ± 4.5	38.1 ± 4.3
Co-trimoxazole intravenous infusion	400	398.91 ± 4.2	400.1 ± 5.1	80	82.8 ± 5.2	81.6 ± 6.1

**Table 4:** Results of the replicate measurements (*n* = 3) for the determination of SMX and TMP in biological fluids.

**SMX**								
**Samples**	**Proposed Method**				**HPLC**			
	**Added** **(µg mL^−1^)**	**Found** **(µg mL^−1^)**	**Recovery** **(%)**	**RSD** **(%, *n* = 3)**	**Added** **(µg mL^−1^)**	**Found** **(µg mL^−1^)**	**Recovery** **(%)**	**RSD** **(%, *n* = 3)**
Serum	10	10.98	109.8	5.54	10	9.3	93.0	6.5
	20	19.10	95.5	6.32	20	19.05	95.2	6.95
	30	32.31	107.7	3.52	30	33.05	110.1	5.32
Plasma	10	9.23	92.3	4.21	10	9.35	93.5	5.62
	20	21.56	107.8	3.11	20	19.32	96.6	4.73
	30	29.11	97.0	4.63	30	32.15	107.2	6.72
**TMP**								
**Samples**	**Proposed Method**				**HPLC**			
	**Added** **(µg mL^−1^)**	**Found** **(µg mL^−1^)**	**Recovery** **(%)**	**RSD** **(%, *n* = 3)**	**Added** **(µg mL^−1^)**	**Found** **(µg mL^−1^)**	**Recovery** **(%)**	**RSD** **(%, *n* = 3)**
Serum	20	20.89	104.4	4.62	20	21.65	108.2	5.97
	10	9.05	90.5	5.31	10	10.21	102.1	4.78
	25	24.32	97.3	4.65	25	23.65	94.6	5.64
Plasma	20	19.52	97.6	3.12	20	19.23	96.15	4.68
	10	10.23	102.3	2.65	10	9.53	95.3	6.28
	25	26.14	104.6	4.77	25	27.15	108.6	6.89

## References

[1] Ma R., Wang Y., Zou X., Hu K., Sun B., Fang W. (2017). Pharmacokinetics of sulfamethoxazole and trimethoprim in Pacific white shrimp, Litopenaeus vannamei, after oral administration of single-dose and multiple-dose. Environ. Toxicol. Pharmacol..

[2] Tungsanga K., Chongthaleong A., Udomsantisuk N., Petcharabutr O.A., Sitprija V., Wong E.C.K. (1988). Norfloxacin versus co-trimoxazole for the treatment of upper urinary tract infections: A double blind trial. Scand. J. Infect. Dis. Suppl..

[3] Cooper J., McGillion F.B. (1978). Treatment of acute exacerbations of chronic bronchitis. A double-blind trial of cotrimoxazole and cephalexin. Practitioner.

[4] Feldman W., Richardson H., Rennie B., Dawson P. (1982). A trial comparing cefaclor with co-trimoxazole in the treatment of acute otitis me. Arch. Dis. Child..

[5] Sayar E., Sahin S., Cevheroglu S., Hincal A. (2010). Development and validation of an HPLC method for simultaneous determination of trimethoprim and sulfamethoxazole in human plasma. Eur. J. Drug Metab. Pharmacokinet..

[6] Ayejuyo O.O., Nwoko C., Hamed M. (2016). Liquid Chromatographic Technique for the Simeltaneous Dtermination of Sulphamethoxazole and Trimethoprim in Pharmaceutical Formulations. UNILAG J. Med. Sci. Technol..

[7] Bedor D.C.G., Gonçalves T.M., Ferreira M.L.L., de Sousa C.E.M., Menezes A.L., Oliveira E.J., de Santana D.P. (2008). Simultaneous determination of sulfamethoxazole and trimethoprim in biological fluids for high-throughput analysis: Comparison of HPLC with ultraviolet and tandem mass spectrometric detection. J. Chromatogr. B.

[8] Da Silva I.S., Vidal D.T.R., Do Lago C.L., Angnes L. (2013). Fast simultaneous determination of trimethoprim and sulfamethoxazole by capillary zone electrophoresis with capacitively coupled contactless conductivity detection. J. Sep. Sci..

[9] Pereira P.F., da Silva W.P., Muñoz R.A.A., Richter E.M. (2016). A simple and fast batch injection analysis method for simultaneous determination of phenazopyridine, sulfamethoxazole, and trimethoprim on boron-doped diamond electrode. J. Electroanal. Chem..

[10] Gonzalez D., Melloni C., Poindexter B.B., Yogev R., Atz A.M., Sullivan J.E., Mendley S.R., Delmore P., Delinsky A., Zimmerman K. (2015). Simultaneous determination of trimethoprim and sulfamethoxazole in dried plasma and urine spots. Bioanalysis.

[11] Sgobbi L.F., Razzino C.A., Machado S.A.S. (2016). A disposable electrochemical sensor for simultaneous detection of sulfamethoxazole and trimethoprim antibiotics in urine based on multiwalled nanotubes decorated with Prussian blue nanocubes modified screen-printed electrode. Electrochim. Acta.

[12] Liu Y.Y., Hu X.L., Bao Y.F., Yin D.Q. (2018). Simultaneous determination of 29 pharmaceuticals in fish muscle and plasma by ultrasonic extraction followed by SPE–UHPLC–MS/MS. J. Sep. Sci..

[13] Amini H., Ahmadiani A. (2007). Rapid and simultaneous determination of sulfamethoxazole and trimethoprim in human plasma by high-performance liquid chromatography. J. Pharm. Biomed. Anal..

[14] Teshima D., Otsubo K., Makino K., Itoh Y., Oishi R. (2004). Simultaneous determination of sulfamethoxazole and trimethoprim in human plasma by capillary zone electrophoresis. Biomed. Chromatogr..

[15] Afkhami A., Khalafi L. (2007). Spectrophotometric investigation of the effect of β-cyclodextrin on the intramolecular cyclization reaction of catecholamines using rank annihilation factor analysis. Anal. Chim. Acta.

[16] Zhang L., Liu Y., Wang Y., Xu M., Hu X. (2018). UV–Vis spectroscopy combined with chemometric study on the interactions of three dietary flavonoids with copper ions. Food Chem..

[17] Esteki M., Nouroozi S., Amanifar S., Shahsavari Z. (2017). A Simple and Highly Sensitive Method for Quantitative Detection of Methyl Paraben and Phenol in Cosmetics Using Derivative Spectrophotometry and Multivariate Chemometric Techniques. J. Chin. Chem. Soc..

[18] Esteki M., Nouroozi S., Shahsavari Z. (2016). A fast and direct spectrophotometric method for the simultaneous determination of methyl paraben and hydroquinone in cosmetic products using successive projections algorithm. Int. J. Cosmet. Sci..

[19] Bro R. (2003). Multivariate calibration: What is in chemometrics for the analytical chemist?. Anal. Chim. Acta.

[20] Othman S. (1990). Multicomponent derivative spectroscopic analysis of sulfamethoxazole and trimethoprim. Int. J. Pharm..

[21] Nevado J.J.B., Gallego J.M.L., Penalvo G.C. (1992). Determination of sulfamethoxazole and trimethoprim by ratio spectra derivative spectrophotometry. Fresenius J. Anal. Chem..

[22] López-Martínez L., López-de-Alba P.L., de-León-Rodríguez L.M., Yepez-Murrieta M.L. (2002). Simultaneous determination of binary mixtures of trimethoprim and sulfamethoxazole or sulphamethoxypyridazine by the bivariate calibration spectrophotometric method. J. Pharm. Biomed. Anal..

[23] Navarro M.V., Cabezón M.A., Damiani P.C. (2018). Simultaneous Determination of Pesticides in Fruits by Using Second-Order Fluorescence Data Resolved by Unfolded Partial Least-Squares Coupled to Residual Bilinearization. J. Chem..

[24] Nikpour H., Mousavi M., Asadollahzadeh H. (2019). Using inclusion complexes for achieving second-order advantage: A novel technique for cinnamic acid derivatives analysis with second-order calibration methods. J. Chemom..

[25] Olivieri A.C. (2005). On a versatile second-order multivariate calibration method based on partial least-squares and residual bilinearization: Second-order advantage and precision properties. J. Chemom..

[26] El-Sheikh A.H., Al-Degs Y.S. (2013). Spectrophotometric determination of food dyes in soft drinks by second order multivariate calibration of the absorbance spectra-pH data matrices. Dye Pigment..

[27] Ho C.N., Christian G.D., Davidson E.R. (1978). Application of the method of rank annihilation to quantitative analyses of multicomponent fluorescence data from the video fluorometer. Anal. Chem..

[28] Hemmateenejad B., Yousefinejad S. (2009). Multivariate standard addition method solved by net analyte signal calculation and rank annihilation factor analysis. Anal. Bioanal. Chem..

[29] Etezadi H., Sajjadi S.M., Maleki A. (2019). Crucial successes in drug delivery systems using multivariate chemometric approaches: Challenges and opportunities. New J. Chem..

[30] Benvidi A., Dadras A., Abbasi S., Tezerjani M.D., Rezaeinasab M., Tabaraki R. (2019). Experimental and computational study of the pKa of coumaric acid derivatives. J. Chin. Chem. Soc..

[31] Alizadeh S., Moghtader M., Aliasgharlou N. (2019). Rank Annihilation Factor Analysis for Spectrophotometric Study of Morphine Based on AuNPs Aggregation Using Multivariate Curve Resolution. Sens. Lett..

[32] Mita H., Mansur U., Sartika J. (2012). Optimation and Validation of Analytical Method of Cotrimoxazole in Tablet and Plasma In vitro by High Performance Liquid Chromatography. J. Bioanal. Biomed..

[33] Bahram M., Mabhooti M. (2009). Rank annihilation factor analysis using mean centering of ratio spectra for kinetic–spectrophotometric analysis of unknown samples. Anal. Chim. Acta.

[34] Meloun M., Čapek J., Mikšík P., Brereton R.G. (2000). Critical comparison of methods predicting the number of components in spectroscopic data. Anal. Chim. Acta.

[35] Abdollahi H., Safavi A., Zeinali S. (2008). Model-based rank annihilation factor analysis for quantitative analysis of mixtures of monoprotic acids using multivariate spectrophotometric acid-base titrations. Chemom. Intell. Lab. Syst..

[36] Zhou W., Moore D.E. (1997). Photosensitizing activity of the anti-bacterial drugs sulfamethoxazole and trimethoprim. J. Photochem. Photobiol. B Biol..

[37] Faber N.M., Buydens L.M.C., Kateman G. (1994). Generalized rank annihilation method. I: Derivation of eigenvalue problems. J. Chemom..

[38] Olivieri A.C., Wu H.-L., Yu R.-Q. (2009). MVC2: A MATLAB graphical interface toolbox for second-order multivariate calibration. Chemom. Intell. Lab. Syst..

[39] Smilde A.K., Tauler R., Saurina J., Bro R. (1999). Calibration methods for complex second-order data. Anal. Chim. Acta.

[40] Rodríguez-Cuesta M., Boqué R., Xavier R.F. (2003). Influence of selectivity and sensitivity parameters on detection limits in multivariate curve resolution of chromatographic second-order data. Anal. Chim. Acta.

[41] Goicoechea H.C., Olivieri A.C. (2005). New Robust Bilinear Least Squares Method for the Analysis of Spectral-pH Matrix Data. Appl. Spectrosc..

[42] Amrhein M., Srinivasan B., Bonvin D., Schumacher M.M. (1996). On the rank deficiency and rank augmentation of the spectral measurement matrix. Chemom. Intell. Lab. Syst..

[43] Keller H.R., Massart D.L. (1991). Evolving factor analysis. Chemom. Intell. Lab. Syst..

[44] Babić S., Horvat A.J.M., Mutavdžić Pavlović D., Kaštelan-Macan M. (2007). Determination of pKa values of active pharmaceutical ingredients. TrAC Trends Anal. Chem..

[45] Chen H., Gao B., Li H., Ma L.Q. (2011). Effects of pH and ionic strength on sulfamethoxazole and ciprofloxacin transport in saturated porous media. J. Contam. Hydrol..

[46] Qiang Z., Adams C. (2004). Potentiometric determination of acid dissociation constants (pKa) for human and veterinary antibiotics. Water Res..

[47] Mikes O., Trapp S. (2010). Acute Toxicity of the Dissociating Veterinary Antibiotics Trimethoprim to Willow Trees at Varying pH. Bull. Environ. Contam. Toxicol..

[48] (2005). Effect of Water Quality on Rejection of Selected Human and Veterinary Antibiotics by Nanofiltration and Reverse Osmosis Membranes—Semantic Scholar. Membr. Technol. Conf..

[49] Cocco L., Roth B., Temple C., Montgomery J.A., London R.E., Blakley R.L. (1983). Protonated state of methotrexate, trimethoprim, and pyrimethamine bound to dihydrofolate reductase. Arch. Biochem. Biophys..

[50] Cao J., Cross R.F. (1995). The separation of dihydrofolate reductase inhibitors and the determination of pKa,1 values by capillary zone electrophoresis. J. Chromatogr. A.

[51] Schwalbe C.H., Cody V., Blair J.A. (1983). Trimethoprim analysis. Proceedings of the 7th International Symposium Structure Study on Antifolate Drugs.

[52] Perrin D. (1965). Dissociation Constants of Organic Bases in Aqueous Solution.

[53] Javier S., Santiago H.-C., Romà T.A., Izquierdo-Ridorsa A. (1999). Procedure for the Quantitative Determination of Mixtures of Nucleic Acid Components Based on Multivariate Spectrophotometric Acid−Base Titrations. Anal. Chem..

